# Community’s perception, experiences and health seeking behavior towards newborn illnesses in Debre Libanos District, North Shoa, Oromia, Ethiopia: Qualitative study

**DOI:** 10.1371/journal.pone.0227542

**Published:** 2020-01-14

**Authors:** Kasahun Girma Tareke, Yohannes Kebede Lemu, Shifera Asfaw Yidenekal, Garumma Tolu Feyissa

**Affiliations:** Department of Health, Behavior and Society, Jimma University, Jimma, Ethiopia; Chinese Academy of Medical Sciences and Peking Union Medical College, CHINA

## Abstract

**Background:**

Worldwide about 4 million newborns die each year; of which around 600,000 newborns die from series bacterial infections. To reduce newborn death, community based newborn care is being implemented in Ethiopia though its utilization by clients is low. Studies conducted to address perception of the community towards newborn illnesses are limited. Therefore, this study was aimed in exploring community member’s perception, experiences and health seeking behavior towards newborn illnesses.

**Methods:**

A descriptive qualitative study was conducted from March 11– April 7, 2019 in Debre Libanos District, Ethiopia. Study participants were recruited purposively from six kebeles and women delivered within two months prior to data collection were the primary study participants. Five in-depth interviews, seven key informant interviews and three focused group discussions were conducted. Data were audio-recorded, transcribed verbatim, translated, and analyzed using inductive thematic analysis in Atlas ti.7.1 software package.

**Result:**

This study found that community members locally diagnose newborn illnesses as sunburn, evil eye, kichitat, megagna, berd, enlarged/dropping of uvula, and common cold from misconceived cause when unspecific types of symptoms are recognized on newborns. For those locally diagnosed newborn illnesses, they primarily prefer traditional medications to manage the illnesses rather than seeking care from health facilities. This study also found that clients seek health care for these newborn illnesses late. They seek care either from traditional or from health facilities when newborns become unable to breast feed, weak and feeling too hot.

**Conclusion:**

Local newborn illness diagnosis negatively affected health seeking behavior of the community members in that they made them to rely on traditional medications or delay in seeking care from health facilities. This might leads to negative consequences like disability and mortality. Therefore, health care providers and policy makers should design social and behavioral change communication (SBCC) to change community member’s health seeking behavior towards newborn illnesses.

## Introduction

Worldwide, about 4 million newborn babies die each year; of which about two-thirds of deaths occur in the first month of life. Eighty-five percent of newborn deaths are due to three main causes: complications of prematurity and low birth weight (LBW), birth asphyxia and infection [1]. Bacterial infection, which is called as Possible serious bacterial infection (PSBI) [1–4], is a cause for an estimated number of 600,000 neonatal deaths per year [2], accounting for approximately 23% of neonatal deaths, yet as high as 50% in low-income settings [5, 6]. Its incidence ranges from 5.5 cases/1,000 live births for blood culture-confirmed infections, to 170 cases/1,000 births for clinically diagnosed cases in community-based settings [7]. In Ethiopia, also it is one cause of newborn mortality [8] and study showed 34.3% neonatal death was caused by neonatal infection [9].

Almost 98% of neonatal deaths due to this infection occur in low- and middle-income countries (LMICs). This is because in resource-limited settings, newborns with signs of PSBI do not receive the recommended inpatient treatment due to limited accessibility, low acceptability or affordability problems resulting in unnecessary infection-related newborn deaths [2]. To overcome these challenges, the World Health Organization [WHO] recommends management of possible serious bacterial infection among newborns at a community level when referral to hospital is not possible [2, 10]. In Ethiopia, this community-based intervention for newborns with PSBI was started between 2008 and 2013, as a pilot to evaluate the impact of a regimen of intramuscular gentamicin and oral amoxicillin, given by health extension workers (HEWs) to newborns and young infants with signs of PSBI when referral is not possible [11] and was launched on March 2013. Currently, it is being implemented as one community-based newborn care (CBNC) package and high impact newborn and child survival intervention [12].

Studies indicated that community-based newborn care interventions like management of PSBI by community health workers is associated with reduced neonatal mortality [6, 11]. However, it was understood that there were gaps in utilizing this service [11, 13]. On the other hand, only limited evidence is available on community’s perception, experience and health seeking behavior towards newborn illnesses. Therefore, this study was focused on qualitative exploration of community’s perception, experiences and health seeking behavior towards newborn illnesses.

## Methods and materials

### Study setting and period

This study was conducted in Debre Libanos District, North Shoa zone, Oromia Regional state, Ethiopia from March 11– April 7, 2019. Debre Libanos District is the second from the least in achieving community-based management of newborn PSBI from North Shoa zone districts [14]. It is located 90 km away from Addis Ababa in northern direction. There is an estimated number of 64, 305 population with 2225 expected number of live births per year who are eligible to community based newborn care [15]. Nearly 77.1% and 22.9% of the population lives in rural and urban, respectively and about 99.29% of the inhabitants practice Ethiopian Orthodox Christianity [16]. Currently, there are two health centers, ten functional health posts [i.e. two urban and eight rural], one nonfunctional health post [with no HEW] and three private primary clinics. Also, there are four Health officers, one BSC nurse, twenty clinical nurses, one public nurse, four laboratory technicians, two druggists, five midwifery nurses, fourteen rural HEWs, and five urban HEWs who provide service for these populations [15].

### Study approach

Descriptive qualitative study design was employed because it is an appropriate design for research questions focusing on discovering who, what, and where of events or experiences happened and it assists in gaining insights from informants regarding a poorly understood phenomenon [17]. Therefore, due to this nature of this study approach, this study was conducted to explore community member’s perception, practice and experiences towards newborn illnesses. This is because there were no studies accessed that was done to explore these issues in this study setting.

### Study participants and participant recruitment

Participants and study sites were selected purposively based on their expected knowledge and rich experience in the community about the community’s day to day experiences related to CBNC. Hence, six kebeles were selected considering the number of catchment kebeles per health center, diversity in their distances from HC, their residence as being rural versus being urban, and performance of HEWs in that setting. Participants recruited and involved on in-depth interviews (IDI) were four women who gave birth within two months prior to data collection and one woman whose newborn was died within two months of life. As key informants, we interviewed one under five clinics focal person, one midwife nurse, HEW, religious leader, kebele chairman, one health center head and one district health office MNCH expert. About 10–12 participants were purposively recruited for each focus group discussion (FGD) and 7–12 individuals participated in the FGDs; with a total of twenty eight participants. The participants recruited and involved in each FGD were women who gave birth within the last two years, women whose newborn was sick and was treated at HC within the last 2–3 years, fathers whose newborn was sick and was treated at HC in the last 2–3 years; husbands, fathers and mothers of women delivered within the last two month prior to data collection; pregnant mothers, mother in laws, mothers who lost their newborn during the first two months of life, father in laws, other married and unmarried males and females found in reproductive age group. Participants from health facilities were recruited based on their role on implementation of the program activities [i.e. as monitor or direct implementer]. At kebele (village) level, study participants were recruited based on their potential of having rich information towards newborn illnesses or their role as a care giver for newborns.

### Data collection procedures (instrument, personnel, data collection)

A total of 12 interviews and three FGDs were conducted with a total of 40 participants; five on IDIs, seven on key informant interviews; and 28 on FGDs comprising diversified group of individuals from different settings using a semi-structured guide. The guides were prepared to cover topics related to a) community’s perception towards newborn illnesses b) community’s experiences towards newborn illnesses, c) community’s health seeking behavior for their sick newborns. The guiding questions were developed in English language in relation to the research questions while taking into account local knowledge and cultural sensitivities. The sequence of the topics generally moved from the more general to the specific questions. Guides were then translated into Afan Oromo and Amharic languages and back-translated into English by an independent translator.

The principal investigator was the modulator throughout the IDIs, key informant interviews and FGDs. At the beginning of the each FGD and IDI, the moderator [principal investigator] explained the purpose of the study and topic of the discussions. Individual-based written informed consent was taken and also consent was obtained to record their voices. The moderator used topic guide to direct the interviews and discussions aiming to cover all relevant topics. In-depth interviews were conducted at their home, interviews conducted with HWs, HEWs, kebele chairman and religious leader were conducted at their office, and FGDs were conducted within their community; means all interviews and FGDs were conducted at their natural settings. The IDIs were conducted one to one in between the principal investigator and participant, but the FGDs were conducted by the principal investigator as a modulator and an assistant as a note taker and audio-recorder. The FGDs were conducted for a time ranging from 1:15 to 1:41 hour and the interviews with community members ranged from 21:33 to 43:51 minute and key informant interviews conducted with health workers ranged from 0:39:40 to 1:12 hour.

### Data analysis

Inductive thematic analysis was employed to analyze the data. The analysis was started in the field debriefing the data with research assistant. Listening the audio-recorded material, verbatim transcription was done by the researcher in support of research assistant. Field notes were incorporated within the transcription simultaneously. Then, the transcriptions were checked for completeness and consistency. After that, all FGD transcripts and transcripts of IDI conducted with health workers were translated from Afan Oromo and Amharic languages to English by the principal investigator. Transcripts of other IDIs and key informant interviews were translated from Afan Oromo and Amharic languages to English by the research assistant. Completeness and consistency of the translations were checked with the transcriptions.

Reading and re-reading of the translations were done to extract important statement from the description and then coding was made line by line. First, the principal investigator and his peer conducted line by line coding (the principal investigator on ATLAS. Ti.7.1 and the assistant manually on Microsoft word) starting with richest data. The codes were then checked for inter-coder consistency and code book manual was developed. Then, the principal investigator coded the whole translations using the code book manual as a guide to ensure code consistency and credibility. Again, the principal investigator coded the whole translations for checking intra-coder consistency. Potential categories and themes were developed by clustering sub-categories and categories, respectively, which answers the research questions. The principal investigator repeated the coding system four times after the first code book was developed while refining the code book, categories and themes. Finally, results were presented with major theme, categories and quotations derived from the data.

### Trustworthiness (rigor)

To keep trustworthiness of the study; to determine how closely study findings reflect and represent the data provided and experienced by participants in relation to the study processes and procedures, the following trustworthiness principles were followed.

### Credibility

Credibility of this study was ensured through peer debriefing, triangulation, member checking, creating rapport and negative case analysis.

#### Peer-debriefing

Two peers were involved on this research process. The first is a research assistant who was involved during data collection, transcription and translation. He was recruited taking into account his experience in assisting qualitative research, knowledge of local language and health background as a criterion. Half day orientation was given to him on the general research process and two IDIs were conducted with him with recently delivered mothers and HEW while pre-testing the IDI guide. The second assistant was the principal investigator colleague who is MPH student. He was involved on the first round coding to develop code book manual.

#### Member checking

At the end of each interview and FGD, the principal investigator summarized and presented major themes to the participants; the principal investigator also discussed on those and some unclear ideas at the end. The transcription and translation were shared and summary of core points and some confusing ideas were presented to HEWs, U-5 clinic focal person, head of health center and district health office maternal, neonatal and child health (MNCH) focal person for clarity and to check the interpretations. Then, they provided their comments and critiques on the raised points and consensus was made on some unclear ideas. The principal investigator also shared the findings of the study with these participants through telephone for checking each quotation mentioned by them to confirm that their ideas have been represented accurately.

#### Triangulation

Method triangulation [IDI and FGD] and data triangulation [data taken from different perspectives (community’s, clients’ and providers ‘perspectives) were combined. During subsequent interviews and focus group discussions, findings from emerging data were briefly presented to participants for confirmation and refutation.

#### Creating rapport

The principal investigator was discussed the importance of the research with zonal health department staffs, district health office staffs, health center staffs and study participants [i.e. spoken with a range of people] and developed relationship, trust and rapport with them. Zonal health department manager and district health officer wrote support letter for the principal investigator and the study participants were recruited together with them. They also supported the principal investigator in facilitating transportation to go kebeles (villages) to conduct IDI and FGDs. Additionally; they regularly discussed the progress of data collection face-to-face and through phone.

#### Negative case analysis

The principal investigator tried to analyze contradicting ideas or deviant cases that emerged in the data by enquiring in-depth information from potential study participants on the consecutive data collection periods.

### Transferability

To ensure transferability, applicability of one set of findings to another setting or the extent to which the reader is able to generalize the findings of a study to her or his own context, the whole research process, participant’s diverse perspectives and experiences, methodology, interpretation of results, and contributions of research assistants were explained clearly through thick description. As it is mentioned on study setting, this study was conducted in Debre Libanos District, North Shoa zone, Oromia Regional state, Ethiopia in which more of its populations are residing at rural, and all most all of them are Ethiopian Orthodox Christian followers. Majority of the population speaks both Amharic and Afan Oromo language although Afan Oromo is predominantly spoken. Most of the kebeles have no transportation service and it has diverse type of topographic nature. Currently, there are two health centers, ten functional health posts [i.e. two urban and eight rural], one nonfunctional health post [with no HEW] and three private primary clinics. Therefore, the data reported here works for settings having similar infrastructure and culture, particularly, the district settings of Ethiopia and other districts with similar settings to what has been described here in other countries.

### Dependability

In order to ensure dependability, the chosen methodology, selection and recruitment of participants, data collection methods and the analysis process were thickly described. Detailed chronology of research activities and processes, memos, data collection and analysis, emerging themes, categories or quotations were audited by advisors, colleagues and audited by other person who has experience of conducting qualitative research to confirm the procedures and verify whether they were used correctly to make both the process and the study output consistent. Thus, with these activities, the process through which findings were derived was made explicit enough.

### Confirmability

Confirmability of this study, whether study’s findings clearly represent participants’ view than the belief, theories or biases of the researcher, was ensured through different techniques.

#### Researcher self-reflectivity and bracketing

The principal investigator is public health officer in his educational back ground that has experience in working at health center with different departments like u-5 clinics, ART clinics, MNCH clinics, etc. The principal investigator also have taken different trainings related to Community Based Newborn Care, including management of newborn possible serious bacterial infection, worked as CBNC focal and participated on different supportive supervisions and PRCMM meetings. HE also has experience on conducting IDIs and FGDs. This preconception knowledge and skills benefited him to set and focus on research questions. The other researchers, who predominantly worked in couching and guiding the principal investigator, had diverse research experiences in community health, evidence-based healthcare and qualitative research. They had educational background of health officer and/or public health with masters and PhD specialties in health promotion and evidence-based healthcare.

The context of this study setting was different from the setting at which the principal investigator have been working on and the participants were also not familiar with him. Therefore, even if bias is inevitable or unavoidable at any studies, the researchers experience and proximity to participants does not lead to a bias that affects the study findings. Rather, it has potentially helped them dig further dependable information from the participants. As much as possible, subjectivity of the principal investigator on this study was managed by balancing the data, analytic processes, and findings in such a way that the reader is able to confirm the adequacy of the findings. Also the principal investigator and assistant know the local language well but the research assistant knows the culture of the community more than the principal investigator. This background has potentially minimized interpretation bias. But, in order not to be overconfident while interpreting the findings, attention was taken also to balance interpretation with direct quotations from study participants. The advisors also supported the researcher during the whole process of the research, for example, during data collection time to include diversified group of participants, monitored the data analysis, and also checked the interpretation of the findings.

#### Audit trial

The findings of this study were audited and verified by advisors, colleagues and other person who has experience in qualitative research. The findings were also verified by key informants like HEW, kebele chairman, and health workers who participated in the study. Moreover, each process was documented and audio records were made available to peer auditor researchers for cross-checking.

### Ethical considerations

Ethical approval was obtained from the Jimma University Research Ethical Review Board, Ethiopia. The right of research participants was maintained by ensuring non-maleficence and underscoring the benefits of the study. Study participants were informed adequately about the purpose of the study, voluntary participation and right to participate or withdraw at any time. In order to ensure their privacy and autonomy, codes were given to participants and participants were informed that the study uses the codes in place of their names in connection to the study findings or in their answers on discussions or interviews. Time was given to them to reflect and provide detailed explanation of the issue. Individual written consents were taken. Separate consents were taken from participants to take their audio-recordings. No participant has refused to participant in the study and to be recorded. All participants have completed the entire sessions of the IDIs and FGDs.

## Result

### Participants’ socio-demographics

Majority of study participants were at age ranged from married females with age ranged from 31–40 years. They were from rural and all of participants were Oromo in ethnicity and follows Ethiopian Orthodox religion. Additionally, majority of them had at least one child.

Demographic characteristics of participants are summarized in Table 1. Mean age was 37.6 years (range: 21–73 years).

**Table 1:** Demographic characteristic of participants in Debre Libanos District, North Shoa, Oromia, Ethiopia, 2019.

Characteristic	Category	N	Characteristic	Category	N
Age	20–30	9	No of children	1	6
	31–40	19		2	4
	41–50	7		3	8
	51–60	3		4	6
	61–70	1		5	4
	>= 71	1		6	2
Sex	Male	16		7	2
	Female	24	Religion	Orthodox	40
Marital status	Single	4	Occupation	House wife	21
	Married	35		Farmers	9
	Widowed	1		Merchant	2
Education status	Illiterate	25		HEW	1
	Primary	14		Health worker	4
	Secondary	4		Kebele chairman	2
	Diploma	6		Priest	1
	Degree	1	Ethnicity	Oromo	40
Residence				Urban	7
				Rural	33

This study found communities perception, experience and health seeking behavior towards newborn illnesses which are organized under two major themes; communities’ perception and experiences towards newborn illness and communities experience on health care seeking decision making for newborn illnesses.

### Community’s perception and experiences towards newborn illnesses

Study participants mentioned that community members locally diagnose newborn illnesses when unspecific symptoms are manifested on newborns. For those illnesses, they primarily use locally prepared traditional medications and home remedies rather than seeking care from health facilities or they seek care from health facilities in case if newborns do not become improved with traditional medications. The common newborn illnesses mentioned by the community members are mentioned as below including their own characteristics, cause, and mode of treatment.

#### Sunburn [locally called Mitch]

Is newborn illness mentioned by participants as being caused by exposure to day time sun light locally termed as *qeter* [from 4–11 o clock], wearing of cloth which stayed on sunlight before cooling it down or when somebody handles the newborn immediately after staying around the fire or on sunlight.

*“Mitch would arise when mothers would go somewhere holding newborns and if she exposes the newborn to sunlight by either suddenly uncovering their clothes or not… when she goes on day time*, *especially from four o clock to eleven o clock …”*

*(42 years old*, *male*, *IDI participant*, *religious leader)*

#### Symptoms of sunburn [Mitch]

They diagnose it when any one or combination of unspecified symptoms like feeling hot, unable to breast feed, vomiting, cough, irritability, weakness, unable to open eye, skin rash, diarrhea, crying, difficulty of breathing, etc. are manifested on newborns.

*“…the symptoms of Mitch are feeling hot*, *body weakness…unable to breast feed*, *unable to open his eye*, *and skin rash …”*

*(42 years old*, *male*, *IDI participant*, *religious leader)*

#### Management of sunburn [Mitch]

Primarily, they manage Mitch using traditional medications prepared from leaf of local herbs like *demakesse (*Ocimum lamiifolium*)*, *bahar zaf (leaf of eucalyptus)*, *kebericho (*Echinops kebericho), *and tunjit (leaf of* Otostegia fruticosa). *Demakesse* is applied externally on the body of the newborn; they provide him/her to drink little by little after punching and diluting with water or steaming the newborn boiling with water or smoking on fire. Additionally, newborns would be steamed using *bahar zaf*, *kebericho and tunjit* by slightly smoking them on fire or boiling with water.

*“When he faces Mitch*, *we make them drink medications such as kebericho*, *tunjit*, *demakesse*, *etc*. *by punching and diluting with water*. *After that*, *they say he would improve*.”

*(34 years old*, *male IDI participant*, *kebele chairman)*

#### Kichitat

Newborn sickness to mean body dislocation, fracture or detachment happening from poor handling of newborns while carrying, breast feeding, etc. or falling down. Participants mentioned if newborns are handled poorly, their lungs, hearts, and intestine could dislocate or detach. Neck of the newborn is the other body part perceived to face fracture or dislocation while newborns become flexed suddenly or when the newborns’ shoulders are suddenly detached.

*“…the other illness there is what we call kichit or warraaqqii*. *In Amharic it is called kichit and in Afan Oromo*, *it is warraaqqi… The lung*, *heart of newborn would move from its original place to other place*. *This happens when they fall to ground or at a time when the mother try to handle the newborn… what we observe on newborns…”*

*(45 years old*, *male FGD participant*, *community member)*

#### Symptoms of kichitat

Study participant mentioned that community members diagnose kichitat when unspecific symptoms like irritability, crying, vomiting, unable to breast feeding, grunting, change of the color of stool to greenish [normally yellow], fast breathing, fever, and cough are manifested on newborns.

*“The symptoms that I saw until now were crying*, *unable to breastfeed*, *becoming irritable…”*

*(28 years old*, *female*, *IDI participant*, *recently delivered mother)*

*“They do not breastfeed*, *and they have fast breathing*, *groan*, *their body feels hot*, *has diarrhea*. *During this time*, *we say that he [newborn] might face Mitch or kichit”*.

*(22 years old*, *female*, *IDI participant*, *recently delivered mothers)*

#### Management of kichitat

Community members reported that they manage newborn illnesses diagnosed as ‘kichitat’ by massaging the body of the suspected of facing fracture, dislocation or detachment using butter or other ointments. They do this for all newborns suspected facing dislocation, fracture or detachment by taking them to traditional bone setter called ‘*wogesha’*. They do not take the newborn to health facility. With this treatment, community members perceive that if their abdomen [intestine] is dislocated, the bone setter would return the abdomen; if their lung is dislocated, the *wogesha* [bone setter] returns their lungs. Then, if the illness does not get improved, the families might give other traditional medications like medications for *Mitch* or take them to health facility.

*“In case of kichit*, *there is local healer who sees this like newborns*. *Since all people cannot do this*, *she massages the body of newborn body using butter gently*. *Then*, *their dislocated body part is returned to its normal site*. *If their abdomen [intestine] is dislocated*, *she returns the abdomen; if their lung is dislocated*, *she returns their lungs*. *This is what they do*.”

*(43 years old*, *male IDI participant*, *kebele chairman)*

*“…The lung*, *heart of newborn would move from original place to other place…During this time*, *she [newborn] would be taken to local traditional healer and healer would return her heart or lung to their normal position*. *This was what we have observed*.”

*(45 years old*, *male FGD participant*, *community member)*

#### Berd

Is another newborn illness mentioned by study participants caused from exposure to cold air at a time when they uncovered their clothes suddenly during cold weather or at a time when they sleep wearing thin clothes.

“There is a so called berd [cold] that occur when newborns become naked their clothes being taken off from them during cold time.”

*(41 years old*, *male*, *FGD participant*, *community member)*

#### Symptoms of berd

Any one or more of unspecified symptoms like cough, fast breathing, crying, unable to breastfeed, irritability, grunting, difficulty of breathing, diarrhea with frequent flatus, chest in drawing, abdominal cramp [to mean diarrhea] would be manifested on newborns.

*“The symptoms that I saw until now were crying*, *unable to breast feed*, *becoming irritable.”*

*(28 years old*, *female*, *IDI participant*, *recently delivered women)*

*“When he [newborn] is exposed to cold*, *there is groan*, *crying*, *cough*, *difficulty of breathing and the like.”*

*(38 years old*, *female*, *IDI participant*, *community member)*

#### Management of berd

Study participants mentioned that there is nothing done for them until their date of baptism [Christianization].

*“…During this time*, *we say that cold has touched him and we cover them with clothes to warm them up*. *… We do not give them anything other than breast feeding until their day of baptism.”*

*(51 years old*, *female*, *FGD participant*, *community member)*

#### Enlargement or dropping of uvula [to mean tonsillitis]

Is newborn illness perceived by community members as dropping of the uvula as a result of dropping of the brain.

“…They [community] perceive as if their brain has dropped when they [newborns] have pain in their throats.”

*(39 years old*, *male*, *FGD participant*, *community member)*

#### Symptoms and management of enlarged or dropped uvula

Study participants mentioned that any one or more of the symptoms like unable to or difficulty of breast feeding, vomiting, fever, body weakness, crying will be present. During this time community members manage it locally by sucking the back side of the newborn neck and or putting locally prepared medications on the head of the newborn with the perception that the brain would return to its normal position which in turn leads to returning of the uvula to its normal position.

*“They [community members] say his throat is enlarged when there is difficulty of breast feeding*, *vomiting and feeling hot*. *During this time*, *they suck back of the newborn’s neck*. *They perceive that at this time the enlarged throat will return to its normal place…*. *Additionally when their throat is painful*, *they become weak and unable to breast feed.”*

*(34 years old*, *male IDI participant*, *kebele chairman)*

*“They might face pain in their throats*. *They just cry*, *unable to breast feed*, *they feel hot … During this time before taking them to health facility…they would put the medication on their head that return the dropped tonsil into its place.”*

*(34 years old*, *female*, *FGD participant*, *community member)*

#### Megagna

Is a newborn illness mentioned by study participants resulted at a time when the devil touches newborns. To prevent this type of newborn illness, community members do not leave the newborn alone or naked, they put metals/sharp things besides them on their sleeping bed or when their body is exposed during night time for breast feeding.

*“In this issue*, *according to our culture*, *there is a belief that something like that of devil might touch them*. *So*, *until priest come to home and clean the home with holy water*, *we place sharp materials above their head*. *If this is not*, *the devil would touch them …”*

*(51 years old*, *female*, *FGD participant*, *community member)*

#### Symptoms of *megagna*

Crying suddenly, paralyzing legs or hands and others symptoms of evil eye sickness.

*“When he [newborn] just becomes suddenly stressed [irritable] and cried*, *we perceive that it [devil] would pinch my newborn.”*

*(34 years old*, *male FGD participant*, *kebele chairman)*

*“…To mean the devil has been touched her according to our community*, *it is when their body becomes rotated or when they cry highly or their legs or hands become flexed or if their necks become rotated …”*

*(34 years old*, *female*, *FGD participant*, *pregnant women)*

#### Management of megagna

Community members treat megagna using traditional medications like steaming medications prepared from local leaf like *tunjit*, and with holy water. But, since most of the time newborns are young and probably have not celebrated their date of baptism, they will not be taken to holy water.

“There is nothing done until they reach their 40 days [males newborn] or 80 days [female newborn].”

*(42 years old*, *male*, *IDI participant*, *religious leader)*

“…we belief it as megagna and we steam tunjit.”

*(42 years old*, *female FGD participant*, *Community member)*

#### Evil eye [locally called buda]

Newborn illness that is perceived to come from exposure to a person possessing a behavior of evil eye. The perception behind this is that, when a person with a behavior of evil eye looks the newborn at any time, they eat them with their internal sprit.

*“…This means that when the person who has that behavior looks at the newborns*, *they eat and pierce with in their internal sprit… This time it is called the newborn was exposed to evil eye.”*

*(28 years old*, *female*, *IDI participant*, *recently delivered women)*

#### Symptoms of evil eye

Any one or combination of unspecific symptoms like unable to breast feed, unable to open their eyes, crying, being irritable, weakness, loss of consciousness, sleeping for a long time [being lethargic], difficulty of breathing, etc.

*“He will face sudden fainting*, *loss of consciousness*, *irritability; he will be lethargic and/or weak.”*

*(28 years old*, *female*, *IDI participant*, *recently delivered women)*

#### Management of evil eye

Community members treat it using traditional medications prepared from substances like *tenadam (leaf of Ruta chalepensis)*, *white onion (root of* Allium sativum), *root of grawa (root of* Withania somnifera), *shiferaw (moringa olifera)*. They might not seek care from health facilities or might seek in case if the newborn not improved with provided traditional medication.

*“…When they think that newborns have faced evil eye sickness*, *they steam medications like tenadam*, *white onion and root of girawa…the illness would be improved when medications provided in terms of steaming*, *smelling or putting on their body… When we say medications for evil eye sickness*, *it would include white onion*, *tenadam and shiferaw…”*

*(42 years old*, *male*, *IDI participant*, *religious leader)*

#### Common cold

Newborn illness which is caused from poor hygienic condition of the newborn cloths, sleeping area, maternal hygienic condition or might be transferred from care giver if they have common cold.

*“…In our perception it will happen from failure to wash their cloths*, *unhygienic condition*….*The mother*, *even before day of epiphany*, *goes to fetch water*, *clean garden*, *and that newborn would stay in unclean area…”*

*(39 years old*, *male FGD participant*, *community member)*

#### Symptoms of common cold

Any one or combination of unspecific symptoms like fever, cough, unable to breast feed, cough, difficulty of breathing, fast breathing, whispering sound, unable to open his eye, grunting, etc.

*“The symptoms that I saw until now were crying*, *unable to breast feed*, *becoming irritable…she [newborn] has cough*, *difficulty of breathing*, *fever…she has fast breathing*, *whispering sound*, *and grunting.”*

*(28 years old*, *female*, *IDI participant*, *recently delivered women)*

#### Management of common cold

Community members treat common cold by letting them drink little by little home based remedies prepared from ginger and tenadam by diluting or mixing it with boiled milk.

*“…for common cold*, *by boiling milk and adding ginger and tenadam*, *I gave to her [newborn] little by little*. *Then*, *little by little she becomes well …”*

*(28 years old*, *female*, *IDI participant*, *delivered mother)*

### The experiences of the community on health care seeking decision making for their sick newborns

Community members decide to seek care for newborn illness either from traditional or modern medication when newborns reach stage of unable to breast feed and when become extremely hot. Additionally, symptoms like grunting, body weakness, difficulty of breathing, frequent vomiting, gasping and change in the skin color to bluish are mentioned by participants as a symptoms warranting them to seek care. They mentioned that care is not sought for newborns that do not have fever or problem of breast feeding. Perceiving the illnesses seen on newborns as simple, not perceiving as care is sought for newborns unless they are unable to breast feed or unless become extremely hot, perception of self-resolution within the next few days or the belief on traditional medicine deters the community members from seeking care till the symptoms worsen.

*“*…*feeling extremely hot and unable to breast feed are the symptoms indicating that newborns would be sick…If they do not feel hot or problem of breast feeding*, *they would not be taken to health facility or would not seek traditional medications for them…”*

*(42 years old*, *male*, *IDI participant*, *religious leader)*

*“…people take them [newborns] when they become severely sick and become weak*. *This means that after trying all their best but not improved*, *for example*, *when he becomes unable to breast feed they take him or her [the newborn] to health facility …”*

*(58 years old*, *male*, *FGD participant*, *community member)*

One U-5 clinic focal person also mentioned that most sick newborns taken to health center for seeking care have general conditions like weakness/lethargy, fast breathing, severe cough, inability to breast feed, high grade fever, etc. This means that community members do not seek care for sick newborns immediately when illness symptoms are seen on newborns.

*“They are weak*, *extremely exhausted*, *and they do not take them immediately when they become sick…first as I have said to you*, *they have fast breathing*, *second they cough highly due to pneumonia; other*, *their temperature becomes high…there are unable to breast feed.”*

*(32 years old*, *male IDI participant*, *U-5 clinic focal)*

## Discussion

This study attempted to explore community’s perception towards newborn illnesses, local newborn diagnosis, local newborn illness management and their perceived diagnosis of illnesses to seek care locally or from health facility.

When someone is exposed to excessive sun light, there is a disease called sun burn manifested with painful, erythematous reaction of the skin with red swelling that is too hot to touch [18]. Unlike this disease, even if participants mentioned sunburn [*Mitch]* is caused from exposure to sunlight or hot environment, the symptoms are different. Rather, symptoms like fever, unable to breast feed, weakness, skin rash, unable to open eye and difficulty of [fast] breathing are part of newborn danger signs [2, 3] and sign and symptoms of PSLBI [1–3]. This implies that it is an illness diagnosed from misconceived cause which needs health education programs like social and behavioral change communications [SBCC] to change the perception of community members on newborn illnesses and their causes. These findings are in agreement with other study conducted in Ethiopia which showed that *Mitch* is a perceived illness from exposure to rays from the sun, especially at mid-day called *Ketir*. The study also reported that community members believed that *Mitch* causes fever, convulsion, red and swollen eyes [19].

The human body part facing dislocation are bones with disruption from joint manifested with abnormally fixed position with loss of normal range of movement in the affected joint. Fracture also has sign and symptoms like pain, swelling, inability to use the injured body part, swelling, bruising, deformity, abnormal movement, etc. This means that in case of both dislocation and fracture, there is visible sign [20]. To the contrary, perception of that newborn lung, heart, intestine, etc. are dislocated without any locally visible sign does not align with sign and symptoms of dislocation or fracture. On the other hand, symptoms like unable to breast feeding, grunting, fast breathing, and fever are parts of newborn danger signs [2, 3] and also signs or symptoms of PSBI [1–3] and the other symptoms are unspecific symptoms that might occur at any illnesses. Therefore, like that of *Mitch*, it is also a perception or local illness diagnosis which might be due to illiteracy, lack of gaining information on newborn danger signs. This underscores the necessity of programs such as social and behavior change (SBCC) programs.

*Berd* is also a perceived newborn illness whose symptoms are not similar with any scientifically diagnosed disease. Nevertheless, its symptoms like fast breathing; unable to breast feed, grunting, difficulty of breathing, and chest in-drawing mentioned by participants are signs of newborn danger signs [2, 3] and also sign and symptoms of PSBI [1–3]. There is also a study that showed *berd* as a local newborn illness from contact to cold air or wind and manifested with fever, difficulty breathing, coughing, sore throat, continuous crying, and inability to feed and open eyes [21]. These findings have similarity on the cause of the illness but the reported symptoms are somewhat different. For example, symptoms like fever, sore throat, continuous crying, unable to open eye are not present on the current study. This might happen due to socio-cultural or geographical differences or due to the day-to-day encounter of different newborn illnesses with different ranges severity.

Enlargement of uvula is a swelling of uvula as a result of infection or trauma. Patients with uvulitis have enlarged or edematous uvula, fever, sore throat, difficulty or pain with swallowing, etc. [22]. To the contrary, our study finding, as reported by study participants, shows that enlargement or dropping of the uvula happens due to descent of the brain and newborns might have sore throat from excessive or prolonged time crying. Thus, there is limited understanding of sore throat among the community. This might be due to low health illiteracy, lack of information on danger signs, lack of BCC, socio-cultural and geographical differences. Further studies may explore the detailed health literacy level of the community and may design tailored interventions. A study has shown that when newborns have fever and when they are unable to feed it is thought to be caused by uvulitis, described as descended uvula or Anker, and tonsillitis or Qimo [19]. The symptoms like unable to or difficulty of breast feeding, vomiting, fever, weakness, etc. are similar with the symptoms mentioned in these studies and are also part of newborn danger signs [2, 3], signs and symptoms of PSBI [1–3]. Therefore, this also needs designing of health education intervention programs that will change the perception of the community members towards newborn illnesses and their causes.

For many African societies, it is an existential reality that illnesses, misfortune and disturbances are almost always attributed to evil spirits [23]. A study also showed that when newborns are in contact with an evil spirit they will develop an illness called *megagna* manifested with swelling of the body, face and hands, peeling off of skin [21]. The current study also found that there is an illness called *megagna* known as a local newborn illness which occurs when devil touches them in case if they [newborns] are left alone, naked or without sharp or metal was placed on their sides. But, the symptoms reported by other studies differ from each other. This might be due to difference in socio-cultural or geographical location or differences on the day to day encounter of the types of illnesses.

Evil eye is perceived to be one cause of illness, accident, misfortunes, or death among communities of different countries and it is perceived to be caused by exposure to people possessing evil spirit. It is socially transmitted from one generation to the next rather than primarily based on scientific findings [23]. Symptoms of illness caused by the evil eye include loss of appetite, excessive yawning, hiccups, vomiting, fever, insomnia, fatigue, depression and diarrhea [24], interruption of breast feeding, lethargy [23]. Studies also showed that babies and children are said to be especially susceptible to harm from the evil eye [19, 20] and it was mentioned that evil eye has power to kill the newborn [25]. Therefore, the finding of both of these studies is consistent from the perspective of the cause and community’s perception of evil eye locally called as ‘buda’ as one newborn illness. But, the perceived symptoms somewhat differ. This might be due to age differences or socio-cultural differences. The symptoms mentioned by the participants of the current study are not specific only to what was reported by the participants as evil eye. For example, symptoms like unable to breast feed, weakness, loss of consciousness, sleeping for long time [lethargic], difficulty of [fast] breathing are also parts of newborn danger signs [2, 3], sign and symptoms of PSBI [1–3]. Therefore, since it is not scientifically proven illness, only perceiving it and confiding in traditional medicine might lead to harmful consequences on newborns. This underscores the importance of health education programs designed to increase the awareness of the community on newborn danger signs.

Common cold is one of acute, self-limiting viral infection of the URT, involving variable degrees of sneezing, nasal congestion and discharge (rhinorrhea), sore throat, cough, low grade fever. In infants, fever and nasal discharge are common manifestations. Additional manifestations may include difficulty of feeding, decreased appetite, and difficulty sleeping [26]. But, the symptoms mentioned by participants like inability to breast feed, difficulty [fast] breathing, and grunting are also parts of newborn danger signs [2, 3], sign and symptoms of PSBI among newborns [1–3]. Since these symptoms are not only unique to common cold, considering it generally as a common cold and not seeking care from health facility might result in complications or even to death. So, this type of community perception might also affect service utilization for newborn PSBI at health post or home level. This warrants the importance of designing appropriate community awareness programs.

The Ethiopian health policy promotes the use of traditional medicine in primary health care delivery system provided that the traditional providers are accredited and licensed by the FMACA [27, 28]. Findings from different studies also showed traditional medicines are being used for promoting, preventing and treating treat different diseases [29, 30]. Community members in this study area use traditional medicine to treat sick newborns and seek care from health facilities if the newborn’s problems are not improved with these medications.

A study conducted at central and southern Ethiopia showed that community members use herbal medicine [e.g. for *Mitch]* and traditional way of treatment using traditional healer like *wogesha* [i.e. to massage the newborn using butter] to treat newborn illnesses [21]. But, the way they seek care for megagna is different from what community members do in other areas. This is because they seek care from health facility [21]. In the current study, community members treat newborns from *megagna* using traditional medicines which might happen due to socio-cultural difference. Even if participants also mentioned holy water is one option for treating newborn illness from *megagna*, they do not use it before the child gets baptized (as per the doctrine of Ethiopian Orthodox Christian). In agreement with this finding, another study reported that it is not allowed to treat sick newborns with perceived newborn danger signs from spiritual causes using holy water before baptism [19].

Study conducted in Nigeria supports this finding; reported that 72% of nursing mothers used herbal medicines for sick neonates and infants, while 100% of the mothers perceived the herbs are efficacious and 96% did not report any adverse outcomes with herbs and 4% of them reported vomiting as a common adverse outcome after using herbs [31]. A study conducted in Bangladesh also showed that one of the barriers for formal health care seeking is a belief that traditional care will cure the illnesses [32]. Study conducted in Ethiopia showed that sick newborns from evil eye, skin rash, and others treated using traditional medicines prepared from local herbs and also by holy water [33].

A study also showed that uvulitis was treated with less conservative traditional treatment by putting leafs on the newborn’s head and applying or rubbing the newborn head so that the uvula that descended into the throat ascends back to its normal position [19]. This is similar with one way of treating enlarged uvula and similar findings were found in this found on this study.

Danger signs are signs that indicate the need for immediate referral or admission to a higher health facility level [1]. This indicates that they are serious life threatening conditions that need attention. Community members decide seeking care for sick newborns after 1–3 days of manifestations such as inability to breast feed, gasping, grunting, extreme hot, and weakness/lethargic, difficulty of [fast] breathing, frequent vomiting and change in the skin color to bluish. These symptoms are part of newborn danger signs [2, 3] and sign and symptoms of PSBI [1–3]. A study conducted at central and southern Ethiopia showed symptoms like continuous crying or irritability, inability to suck, fever, vomiting, diarrhea, stomach ache, difficult or fast breathing are used as a signs of newborn illness for seeking care either from traditional or health facilities [21]. Another study also showed that mothers’ stories of newborn illness started with a baby struggling to suck or when refusing to take breasts milk. Mothers who sought health-care typically identified three signs of severe illness: not breastfeeding, difficulties in breathing, and fever. They stayed until these symptoms happened considering the problem as only minor and waited at home to see if the child gets better [34]. However, newborns with these signs or symptoms might not be treated at health post; for example, newborns with inability to breast feed at all need mandatory referral to higher health facilities for advanced management [2]. Perceiving illnesses seen on newborns as simple, perceiving that newborns do not get sick unless they are unable to breast feed or have high grade fever, the perception that illnesses resolve themselves within the next few days or belief in traditional medicine were the reasons mentioned by participants to stay until this time. This indicates that community members delay seeking care for sick newborns. Study conducted in Baghdad indicated that mothers who perceived illness in newborns delayed seeking advice or treatment outside the home or did not take newborns to the health facilities because they expect self-resolution of the illness [35]. Such perceptions might happen from the lack of information on danger signs during ANC, delivery, PNC, etc.; absence of pregnant women conference, failure to attend ANC, lack of HEWs committed to provide PNC, etc.

## Conclusion

This study found that community members diagnose newborn illnesses from misconceived causes, even if the symptoms mentioned by study participants are parts of newborn danger signs and sign and symptoms of PSBI. This perception leads the community members to develop perception in that considering newborns illnesses as simple one that resolves by self within few days, considering such illnesses as they have no treatment at health facilities and made them to rely on traditional medications rather than seeking care from health facilities. The study also reveals that community members decide seeking care for their sick newborns late after newborns develop manifestations of danger signs. These negatively affected health seeking behavior of the community members towards newborn illnesses, especially relying on traditional medicine and late seeking care. This by itself might leads to negative consequences like disability and mortality. On the other hand, only preference on traditional medicine might also leads to drug toxicities, complications from delaying care seeking from health facilities, which finally leads to disability and or death. This underscores the importance of taking action to develop strategies to change the perception of these community members towards newborn illnesses. Therefore, health care providers and policy makers should design and conduct social and behavioral change communication programs to change the perception of the community with regard to health seeking behavior for newborn illnesses from health facilities as early as possible.
